# Anxiety and Emotional Intelligence as Predictors of Coping with Stress in Patients with Personality Disorders—A Single-Arm Pre–Post Observational Study

**DOI:** 10.3390/jcm15041583

**Published:** 2026-02-17

**Authors:** Marta Furman, Aleksandra Gradowska, Katarzyna Bliźniewska-Kowalska, Justyna Kunikowska, Małgorzata Gałecka

**Affiliations:** 1 Specialized Psychiatric Health Care Center, J. Babiński Hospital, 91-229 Lodz, Poland; fuurman.m@gmail.com; 2 Department of Psychological Research Methodology, SWPS University of Social Sciences and Humanities, 03-815 Warsaw, Poland; ola.gradowska@gmail.com; 3 Department of Adult Psychiatry, Medical University of Lodz, 91-229 Lodz, Poland; katarzyna.blizniewska-kowalska@umed.lodz.pl (K.B.-K.); justyna.kunikowska@umed.lodz.pl (J.K.); 4 Department of Psychotherapy, Medical University of Lodz, 91-229 Lodz, Poland

**Keywords:** anxiety, emotional intelligence, coping with stress, personality disorders

## Abstract

**Background**: The aim of this study was to examine the relationship between anxiety levels, emotional intelligence, and stress coping strategies in individuals diagnosed with personality disorders. According to Lazarus and Folkman’s transactional model of stress, the appraisal of stressors and available psychological resources determines the selection of coping strategies—whether adaptive or maladaptive. **Material and Methods**: This observational case series study involved 30 individuals diagnosed with personality disorders (ICD-10 codes F60 and F61). Psychological assessments were conducted at two time points: upon admission to a day-care psychiatric unit and after three months of standard therapeutic intervention. The following standardized instruments were administered: the State-Trait Anxiety Inventory (STAI), the Emotional Intelligence Questionnaire (INTE), and the Mini-COPE Inventory for Coping with Stress. **Results**: Elevated levels of anxiety—particularly trait anxiety—were significantly associated with maladaptive coping strategies, including denial and self-blame. Conversely, higher emotional intelligence was positively correlated with the use of adaptive coping mechanisms, such as planning and proactive problem-solving. **Conclusions**: The findings support the hypothesis that both anxiety and emotional intelligence are significant predictors of stress coping styles in individuals with personality disorders. The results underscore the importance of considering these psychological variables in the design and implementation of therapeutic programs. Enhancing emotional intelligence may substantially improve treatment outcomes and overall psychological functioning in this clinical population. However, further studies with larger sample sizes are needed.

## 1. Introduction

For many years, clinical psychology and empirical psychological research have sought to analyze the interplay between anxiety, emotional intelligence, and coping strategies in the face of stress. These three psychological constructs do not function in isolation but form a dynamic system of interrelated mechanisms that influence the effectiveness of an individual’s adaptation to psychological challenges [1,2,3,4].

Anxiety, as a predictor of coping styles, manifests in two fundamental forms: as a trait—denoting a stable tendency to experience anxiety—and as a state—referring to a transient emotional response to a specific situation [5]. According to Marakshina et al. (2025) individuals with elevated anxiety levels are more inclined to employ emotion-focused and avoidant coping strategies, such as emotional suppression, social withdrawal, and rumination [6]. Over time, these maladaptive strategies may exacerbate emotional distress and contribute to the deterioration of psychological functioning. In contrast, individuals with lower anxiety levels are more likely to engage in problem-focused (i.e., solution-oriented) coping strategies, such as seeking instrumental support, action planning, or positively reappraising the situation [7]. Such approaches facilitate the development of psychosocial resources and foster psychological resilience [8].

Emotional intelligence, as conceptualized by Mortillaro et al. (2023) encompasses the capacity to perceive, understand, and regulate emotions—both one’s own and those of others [9]. Meta-analyses conducted by Chen et al. (2025) and Povell et al. (2024) have demonstrated a robust association between high levels of emotional intelligence and the frequent use of adaptive coping mechanisms, including positive reframing, active coping, seeking social support, and acceptance [10,11]. Individuals with high emotional intelligence are more adept at identifying their emotional states, attributing appropriate meaning to them, and managing them constructively. In the context of stress, this emotional competence is associated with reduced susceptibility to maladaptive reactions such as catastrophic thinking, impulsivity, substance misuse, and aggression [2].

Importantly, emotional intelligence may serve as a buffer against the detrimental effects of anxiety [1]. Even in the presence of elevated anxiety, individuals with high emotional intelligence may avoid maladaptive coping by interrupting the cycle of stressor–reaction–avoidance through effective emotional regulation. This interaction suggests a moderating model in which anxiety influences stress responses, but its impact may be attenuated by high levels of emotional intelligence. In other words, the ability to recognize and regulate emotions mitigates the negative effects of anxiety, resulting in milder consequences of chronic stress and preserving psychological functioning.

Patients diagnosed with personality disorders often exhibit significant deficits in emotional regulation and heightened levels of anxiety [12]. Souza et al. (2024) report that individuals with borderline personality disorder, in particular, tend to display low emotional intelligence and high anxiety, which contributes to frequent emotional crises [13]. Such patients are more vulnerable to impulsive behaviors, self-harm, and escalating aggression. Clinical studies have confirmed that among individuals with borderline personality disorder, low emotional intelligence is associated with elevated anxiety and the use of avoidant or self-harming coping strategies. Conversely, those with higher emotional intelligence tend to seek therapeutic or familial support, which positively correlates with more favorable therapeutic outcomes [11].

Emotional intelligence plays a pivotal role in the treatment of personality disorders. Patients with higher emotional intelligence are more likely to form therapeutic alliances effectively, comprehend the objectives of therapy, and utilize cognitive-behavioral techniques with greater efficacy [14]. In contrast, low emotional intelligence may represent a barrier to progress, necessitating the incorporation of emotional skills training into the psychotherapeutic process. Interventions such as Dialectical Behavior Therapy (DBT), Mentalization-Based Therapy (MBT), Acceptance and Commitment Therapy (ACT), and Schema Therapy not only target anxiety and impulsivity but also foster emotional awareness and the development of adaptive coping mechanisms. These benefits are supported by numerous clinical trials and meta-analyses [14,15].

**Figure 1:**
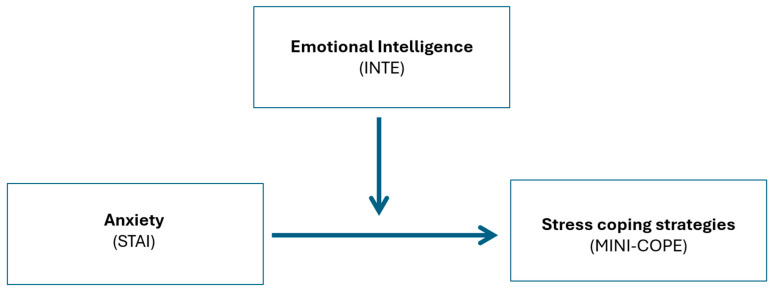
Model of the relationship between anxiety (risk variable), emotional intelligence (moderating variable), and stress coping strategies (outcome variable). Methods of measurement are indicated in brackets.

The theoretical basis for this work is grounded in Lazarus and Folkman’s transactional model of stress, which posits that the appraisal of stressors and available psychological resources determines the selection of coping strategies—either adaptive or maladaptive. On the basis of the evidence reviewed above, a statistical model may be proposed in which anxiety functions as a risk factor, emotional intelligence serves as a moderating variable, and stress coping outcomes are the dependent result of the interaction between the two [1] (see Figure 1). Such a model allows for the quantification of the extent to which emotional intelligence mitigates the adverse impact of anxiety and predicts the types of coping strategies employed in varying psycho-emotional configurations. Furthermore, this model can inform diagnostic assessment and the planning of therapeutic interventions aimed at enhancing emotional intelligence and reducing anxiety in patients with personality disorders (Figure 1).

The aim of this study was to examine the relationship between anxiety levels, emotional intelligence, and stress coping strategies in individuals diagnosed with personality disorders. The research hypotheses were as follows:

- Individuals with higher levels of anxiety are more likely to use maladaptive stress coping strategies.
- Higher levels of emotional intelligence correlate with more adaptive coping strategies.
- Emotional intelligence moderates the relationship between anxiety and stress coping strategies (Figure 1).
- A reduction in state anxiety and selective changes in coping strategies over the 3-month intervention period, whereas trait anxiety and emotional intelligence were expected to be relatively stable.

## 2. Materials and Methods

Patients’ Information: The study sample comprised 31 individuals diagnosed with personality disorders, classified according to ICD-10 criteria (F60 and F61), who were receiving care at a day treatment unit specializing in neurotic and personality disorders. All the participants were adults deemed eligible for psychotherapeutic intervention. The study group included 22 women and nine men, aged between 19 and 50 years, with a mean age of 32.06 years. To enhance transparency and completeness of reporting, we followed general reporting principles for observational pre–post designs, and supplemented them with selected CARE items (see Supplementary Materials) where applicable (e.g., clinical setting, intervention description, timeline, and outcomes) [16,17].

Therapeutic Intervention: During treatment, patients undergo, over a three-month period, group psychotherapy at a frequency of 3 times per week for 3 h each, as well as psychoeducational interventions 2 times per week, and once per week a one-hour individual psychotherapeutic session supporting the group psychotherapy process. Patients continue their psychiatric treatment process and applied psychotherapy during this time. All of the above-described therapeutic methods are recognized standard treatment methods in day care units, and the study was observational in nature.

Psychotherapy provided within the day care unit was conducted using a psychodynamic approach. This approach assumes that the symptoms currently experienced and difficulties in emotional and interpersonal functioning are related to unconscious internal conflicts, established relationship patterns, and ways of regulating emotions, shaped in the course of earlier developmental experiences. The therapeutic work focused on exploring the patients’ emotional experiences, their ways of responding in relationships with others, and the meanings they attached to their own experiences, while taking into account the dynamics of the therapeutic relationship. The aim was to increase insight, improve the ability to understand one’s own emotional states, and gradually modify maladaptive patterns of functioning.

From the patient’s perspective, participation in three months of daily therapy was primarily associated with a change in the way they experienced and regulated anxiety and responded to stressful situations. Although the overall tendency to experience anxiety remained relatively constant, the daily intensity of emotional tension decreased, which translated into a greater sense of control in everyday situations.

Patients were able to experience less emotional reactivity and were less likely to react impulsively when faced with difficult events. Another noticeable change was a reduction in the use of self-critical and self-blaming strategies, which had previously been the dominant way of coping with tension.

After completing therapy, patients more often resorted to more constructive forms of response, such as seeking emotional support, using humor, or referring to meaningful and reflective resources.

From the perspective of everyday functioning, this could mean greater openness in interpersonal relationships, less tendency to withdraw, and greater willingness to share difficulties with others. At the same time, therapy did not lead to a clearly noticeable change in basic emotional competencies, such as understanding or regulating emotions, which suggests that this process requires more time and more in-depth work.

From the patient’s perspective, the therapy could be perceived as intense and demanding, but at the same time conducive to a gradual shift from avoidance-based and self-critical responses to more adaptive, supportive ways of coping with everyday stress.

Timeline and Diagnostic Assessment: Psychological assessments were conducted at two time points: upon admission to the day-care psychiatric unit and after three months of therapeutic intervention. The following standardized instruments were administered: the State-Trait Anxiety Inventory (STAI), the Emotional Intelligence Questionnaire (INTE), and the Mini-COPE Inventory for Coping with Stress.

The State-Trait Anxiety Inventory (STAI) is used to measure anxiety as a state (situational) and a trait (a constant tendency to feel anxious). It comprises two independent subscales, each containing 20 items. Respondents evaluate the intensity of their feelings using a four-point scale. In the present study, primary emphasis was placed on the trait anxiety subscale, given the focus on relatively stable emotional tendencies that may influence coping styles.

The Emotional Intelligence Scale (INTE) was employed to assess levels of emotional intelligence, operationalized in accordance with Mortillaro et al. (2023) theoretical model [9]. This model highlights the capacity to perceive, understand, and regulate emotions in oneself and in others. The INTE comprises 33 statements, with responses recorded on a five-point scale ranging from “definitely no” to “definitely yes”.

To evaluate stress coping strategies, the Mini-COPE—an abbreviated version of the COPE Inventory—was utilized. In its Polish adaptation, the instrument consists of 28 items grouped into 14 subscales, each corresponding to a specific coping strategy (e.g., active coping, denial, humor, religion, self-blame, self-distraction). Respondents indicate the frequency with which they employ each strategy in stressful situations using a four-point scale. Particular attention was given to strategies typically classified as adaptive (e.g., active coping, planning, seeking instrumental and emotional support) versus maladaptive (e.g., self- blame, self- distraction, denial, substance use, etc.).

## 3. Results

### 3.1. Are People with Higher Levels of Anxiety More Likely to Use Maladaptive Stress Coping Strategies?

To examine the relationship between anxiety levels and maladaptive coping strategies, before therapy, Pearson correlation analyses were conducted and supplemented with 95% bias-corrected and accelerated (BCa) bootstrap confidence intervals based on 1000 resamples.

The results indicated that trait anxiety (STAI-X2) was significantly and positively associated with several maladaptive coping strategies. Specifically, trait anxiety showed a moderate positive correlation with denial (*r* = 0.421, *p* = 0.020, 95% BCa CI [0.150, 0.624]). The confidence interval did not include zero, indicating a stable association. In addition, trait anxiety was significantly correlated with self-blame (*r* = 0.524, *p* = 0.003, 95% BCa CI [0.139, 0.737]) and behavioral disengagement (*r* = 0.405, *p* = 0.026, 95% BCa CI [0.063, 0.704]), with both relationships demonstrating moderate effect sizes and confidence intervals that excluded zero.

No significant association was observed between trait anxiety and substance use (*r* = 0.158, *p* = 0.404, 95% BCa CI [−0.208, 0.440]). Similarly, the correlation between trait anxiety and self-distraction did not reach statistical significance (*r* = −0.273, *p* = 0.144), and the corresponding confidence interval (95% BCa CI [−0.550, −0.017]), suggesting that this relationship was not stable in the present sample.

In contrast, state anxiety (STAI-X1) was not significantly correlated with any of the maladaptive coping strategies examined (all *p* values > 0.05). For all associations involving state anxiety, the 95% BCa confidence intervals encompassed zero, indicating a lack of reliable relationships between current anxiety levels and maladaptive coping strategies.

Additional analyses revealed significant associations among coping strategies themselves. Self-distraction was negatively correlated with self-blame (*r* = −0.420, *p* = 0.021, 95% BCa CI [−0.652, –0.121]), indicating that greater use of self-distraction was associated with lower levels of self-blame. Self-distraction was also negatively correlated with behavioral disengagement (*r* = −0.437, *p* = 0.016); however, the corresponding confidence interval marginally included zero (95% BCa CI [−0.750, 0.033]), suggesting that this relationship should be interpreted with caution.

To examine the associations between anxiety levels and maladaptive coping strategies after therapy (Time 2), Pearson correlation analyses were conducted and supplemented with 95% bias-corrected and accelerated (BCa) bootstrap confidence intervals based on 1000 resamples.

The results indicated that trait anxiety assessed after therapy (STAI-X2, Time 2) remained significantly and positively associated with several maladaptive coping strategies. Specifically, trait anxiety showed a strong positive correlation with behavioral disengagement (*r* = 0.739, *p* < 0.001, 95% BCa CI [0.494, 0.894]), indicating that individuals with higher post-treatment trait anxiety were more likely to disengage behaviorally when facing stress. Trait anxiety was also significantly correlated with self-blame (*r* = 0.610, *p* < 0.001, 95% BCa CI [0.312, 0.804]) and denial (*r* = 0.395, *p* = 0.031, 95% BCa CI [0.102, 0.660]), with all confidence intervals excluding zero, suggesting stable and moderate-to-strong associations.

No statistically significant association was observed between post-treatment trait anxiety and substance use (*r* = 0.223, *p* = 0.235, 95% BCa CI [−0.271, 0.524]) or self-distraction (*r* = 0.266, *p* = 0.155, 95% BCa CI [−0.080, 0.560]), as the corresponding confidence intervals included zero.

With regard to state anxiety after therapy (STAI-X1, Time 2), significant positive correlations were observed with behavioral disengagement (*r* = 0.681, *p* < 0.001, 95% BCa CI [0.379, 0.851]) and self-blame (*r* = 0.432, *p* = 0.017, 95% BCa CI [0.114, 0.672]). These findings indicate that higher levels of situational anxiety following therapy were associated with greater reliance on disengagement-based and self-critical coping strategies. In contrast, state anxiety was not significantly related to substance use, self-distraction, or denial (all *p* values > 0.05), and the corresponding confidence intervals encompassed zero.

Additional analyses revealed strong intercorrelations among maladaptive coping strategies at post-treatment. Behavioral disengagement was positively associated with self-blame (*r* = 0.613, *p* < 0.001, 95% BCa CI [0.383, 0.781]), self-distraction (*r* = 0.553, *p* = 0.002, 95% BCa CI [0.302, 0.752]), and denial (*r* = 0.406, *p* = 0.026, 95% BCa CI [0.083, 0.657]), indicating a coherent cluster of disengagement-oriented coping responses. Furthermore, self-distraction was significantly correlated with self-blame (*r* = 0.409, *p* = 0.025, 95% BCa CI [0.130, 0.689]) and denial (*r* = 0.490, *p* = 0.006, 95% BCa CI [0.132, 0.775]), suggesting that these strategies tended to co-occur after therapy.

To complement the correlational analysis, participants were divided into low- and high-anxiety groups (based on a median split), and although median-split procedures reduce statistical power and artificially dichotomize continuous variables, we used this exploratory approach to illustrate potential group differences, examining whether these groups differed in their use of maladaptive coping strategies. The median split was used solely for exploratory descriptive purposes to illustrate potential group differences. All inferential conclusions are based on continuous-variable analyses. Following previous research, denial, behavioral disengagement, self-blame, and substance use were classified as maladaptive coping strategies, whereas active coping, planning, acceptance, positive reframing, and seeking social support were considered adaptive. Self-distraction was treated as a potentially maladaptive strategy due to its avoidant nature.

To complement the correlational analyses, exploratory group comparisons were conducted using median splits to examine differences in maladaptive coping strategies between individuals with low versus high levels of anxiety before and after therapy. Prior to treatment, neither state anxiety (STAI-X1, Time 1) nor trait anxiety (STAI-X2, Time 1) differentiated participants with respect to maladaptive coping strategies, as no significant group differences were observed across denial, self-distraction, self-blame, substance use, or behavioral disengagement (all *ps* > 0.05).

After therapy, individuals with low versus high state anxiety (STAI-X1, Time 2) did not differ significantly in denial, self-distraction, self-blame, or substance use (all *ps* > 0.05); however, a significant difference emerged for behavioral disengagement, with higher state-anxiety participants reporting greater use of this strategy (*U* = 57.00, *Z* = −2.23, *p* = 0.026, exact *p* = 0.031), corresponding to a medium-to-large effect size (*r* = −0.41).

A clearer differentiation was observed for trait anxiety after therapy (STAI-X2, Time 2). Individuals with higher trait anxiety reported significantly greater use of self-blame (*U* = 53.50, *Z* = −2.54, *p* = 0.011, exact *p* = 0.013, *r* = −0.46), reflecting a medium-to-large effect, as well as behavioral disengagement (*U* = 38.00, *Z* = −3.17, *p* = 0.002, exact *p* = 0.001), indicating a large effect (*r* = −0.58). No significant differences were found for denial, self-distraction, or substance use (all *p* > 0.05).

Overall, these findings indicate that although therapy may attenuate anxiety-related differences in several maladaptive coping strategies, self-blame and behavioral disengagement remain selectively elevated among individuals with persistently high trait anxiety, suggesting that these strategies reflect more stable, personality-linked coping tendencies that are less responsive to short-term therapeutic change.

### 3.2. Do Individuals with Higher Emotional Intelligence Use More Adaptive Coping Strategies?

Next, the hypothesis that higher levels of emotional intelligence are associated with more frequent use of adaptive coping strategies was examined using Pearson’s *r* correlations. INTE questionnaire scores were correlated with indicators of adaptive coping strategies. Given the large number of correlations examined, the risk of inflated Type I error was substantial. No correction for multiple comparisons (e.g., Holm–Bonferroni or false discovery rate procedures) was applied, in line with the exploratory nature of the analyses. The primary aim of the analysis was to identify patterns of associations and generate hypotheses rather than to conduct strict confirmatory testing. In exploratory research, the use of conservative correction procedures often leads to an excessive increase in Type II error. Given the limited statistical power, the application of such corrections—particularly Holm–Bonferroni—would have substantially reduced the ability to detect effects of moderate magnitude which, although potentially unstable, may be of theoretical and clinical relevance. Rather than relying solely on statistical significance, the results were supplemented with effect sizes and 95% bootstrap confidence intervals (BCa), allowing for the evaluation of both the strength and precision of the estimates independently of *p*-values. Consequently, borderline *p*-values (e.g., *p* = 0.040–0.060) and isolated significant findings should be interpreted with particular caution.

Adaptive coping strategies included active coping, planning, positive reframing, acceptance, seeking instrumental and emotional support, and humour, reflecting task-oriented and constructive approaches to stress management as defined in the Mini-COPE inventory.

At baseline (pre-treatment), Pearson’s *r* correlations conducted prior to therapy indicated that emotional intelligence was positively associated with the use of adaptive coping strategies, particularly those reflecting problem-focused, cognitive, and social coping.

Overall emotional intelligence (INTE total score). The total score of emotional intelligence was positively and significantly correlated with active coping (*r* = 0.533, *p* = 0.002; 95% BCa CI [0.18, 0.75]), planning (*r* = 0.624, *p* < 0.001; 95% BCa CI [0.37, 0.81]), seeking emotional support (*r* = 0.488, *p* = 0.006; 95% BCa CI [0.15, 0.75]), and positive reframing (*r* = 0.533, *p* = 0.002; 95% BCa CI [0.17, 0.77]). These associations suggest that individuals with higher emotional intelligence were more likely to engage in active problem-solving efforts, cognitive reappraisal, and the use of interpersonal resources when coping with stress.

No statistically significant associations were observed between overall emotional intelligence and acceptance (*p* = 0.055) or humor (*p* = 0.789).

Factor I of emotional intelligence (emotion recognition and understanding) showed a consistent pattern of moderate to strong positive associations with key adaptive coping strategies. Significant correlations were found with active coping (*r* = 0.679, *p* < 0.001; 95% BCa CI [0.45, 0.81]), planning (*r* = 0.693, *p* < 0.001; 95% BCa CI [0.48, 0.85]), positive reframing (*r* = 0.611, *p* < 0.001; 95% BCa CI [0.27, 0.81]), seeking emotional support (*r* = 0.499, *p* = 0.005; 95% BCa CI [0.13, 0.79]), and seeking instrumental support (*r* = 0.466, *p* = 0.010; 95% BCa CI [0.11, 0.72]).

This pattern indicates that individuals who are more capable of accurately recognizing and understanding their emotional states tend to adopt more constructive, goal-oriented, and socially engaged coping strategies. The association between Factor I and acceptance did not reach statistical significance (*p* = 0.065), and humor was not related to this dimension of emotional intelligence.

In contrast, Factor II of emotional intelligence (emotion regulation) demonstrated a weaker and less consistent pattern of associations with adaptive coping strategies. Significant relationships were observed only for planning (*r* = 0.470, *p* = 0.009; 95% BCa CI [0.11, 0.70]) and for the overall emotional intelligence score. No significant correlations emerged between Factor II and other adaptive strategies, including active coping, social support seeking, positive reframing, acceptance, or humor.

Taken together, these findings suggest that baseline adaptive coping was more strongly linked to the ability to recognize and understand emotions (Factor I) than to emotion regulation abilities (Factor II). This pattern implies that emotional awareness and insight may play a particularly important role in facilitating constructive coping responses, even prior to therapeutic intervention, whereas emotion regulation skills appear to be less directly related to coping strategy selection at baseline (Table 1).

A similar comparison was conducted using data collected after the therapeutic intervention, where noticeably fewer statistically significant associations were observed. The correlations identified were positive in a direction that was predominantly positive, but weaker in strength compared to the previous analysis (Table 2). This pattern suggests a post-treatment shift toward more selective and structured coping responses. Specifically, associations between emotional intelligence and cognitively oriented strategies (planning, positive reframing) remained stable, whereas links with more spontaneous or socially mediated strategies (active coping, seeking emotional support) tended to attenuate. One plausible explanation is that therapeutic intervention reduced interindividual variability in these behaviors by promoting standardized coping techniques, thereby weakening correlations driven by individual differences.

Pearson’s *r* correlations conducted after therapy indicated that emotional intelligence remained positively associated with the use of selected adaptive coping strategies; however, the overall pattern of associations was more differentiated than that observed prior to treatment. In contrast to baseline findings, post-therapy coping appeared to be more strongly linked to planning and cognitive restructuring strategies, while associations with social support and active coping were attenuated. The total emotional intelligence score was positively and significantly correlated with planning (r = 0.633, *p* < 0.001; 95% BCa CI [0.31, 0.84]), positive reframing (r = 0.527, *p* = 0.003; 95% BCa CI [0.23, 0.77]), and acceptance (r = 0.398, *p* = 0.029; 95% BCa CI [0.06, 0.63]). These associations indicate that individuals with higher overall emotional intelligence after therapy were more likely to engage in goal-oriented cognitive coping, reinterpret stressful experiences in a more adaptive manner, and accept stressors that could not be changed.

The association between overall emotional intelligence and active coping did not reach statistical significance (*p* = 0.056), and no significant relationships were observed for seeking instrumental support, seeking emotional support, or humor.

Factor I of emotional intelligence (emotion recognition and understanding) demonstrated a robust pattern of positive associations with key adaptive coping strategies following therapy. Significant correlations were observed with active coping (*r* = 0.443, *p* = 0.014; 95% BCa CI [0.15, 0.66]), planning (*r* = 0.571, *p* < 0.001; 95% BCa CI [0.29, 0.76]), positive reframing (*r* = 0.597, *p* < 0.001; 95% BCa CI [0.33, 0.79]), and acceptance (*r* = 0.340, *p* = 0.066; marginal).

This pattern suggests that individuals who are better able to recognize and understand emotional states continue to preferentially employ deliberate, cognitively mediated coping strategies, even after therapeutic intervention. In contrast, Factor I was not significantly associated with social support seeking or humor.

Factor II of emotional intelligence (emotion regulation) showed a more selective and distinct pattern of associations after therapy. Significant positive correlations were found with planning (*r* = 0.527, *p* = 0.003; 95% BCa CI [0.10, 0.79]) and acceptance (*r* = 0.402, *p* = 0.028; 95% BCa CI [0.00, 0.67]). No significant associations emerged for active coping, social support seeking, positive reframing, or humor.

These findings indicate that post-therapy emotion regulation abilities are particularly linked to structured, goal-directed coping and acceptance-based strategies, rather than to emotionally expressive or socially oriented forms of coping.

Taken together, the post-therapy results suggest a shift in the functional role of emotional intelligence in coping processes. While emotion recognition and understanding (Factor I) remained broadly associated with multiple adaptive coping strategies, emotion regulation (Factor II) became more specifically linked to planning and acceptance, suggesting a refinement rather than a generalization of coping skills following therapy.

Compared to baseline, adaptive coping after therapy appears less reliant on broad emotional awareness alone and more strongly grounded in intentional regulation and cognitive organization of stress responses. This pattern may reflect therapeutic gains that support the translation of emotional insight into more focused, self-regulatory coping behaviors.

### 3.3. Does Emotional Intelligence Moderate the Relationship Between Anxiety and Coping Style? (Model Verification)

A series of moderated regression analyses (PROCESS Model 1) were conducted to examine whether emotional intelligence (EI) moderates the relationships between anxiety (state and trait) and avoidance-oriented coping strategies (self-blame, behavioral disengagement, denial, and self-distraction). Analyses were performed separately for data collected before therapy (T1) and after therapy (T2).

The selection of anxiety (state and trait), emotional intelligence, and avoidance-oriented coping strategies was theoretically grounded in the transactional model of stress and coping [18,19,20], which conceptualizes anxiety as a determinant of cognitive–emotional appraisal and coping responses, and personal resources as factors that may modify stress reactivity. Emotional intelligence was therefore examined as a potential moderator specifically for coping strategies that are strongly emotion-driven, avoidance-oriented, and consistently associated with anxiety in prior research. Based on these criteria, self-blame, behavioral disengagement, denial, and self-distraction were selected for moderation analyses, as they represent maladaptive or potentially dysregulated forms of coping linked to heightened anxiety and emotion regulation difficulties. In contrast, adaptive strategies such as active coping, planning, acceptance, and seeking social support were excluded, as the literature indicates that they are more strongly determined by cognitive and contextual resources, with emotional intelligence functioning primarily as a direct predictor rather than a moderator. Moderation analyses were conducted using PROCESS Model 1 and were exploratory and hypothesis-generating rather than confirmatory.

Pre-therapy assessment (T1)

At Time 1, regardless of anxiety type (state vs. trait), no significant moderating effects of emotional intelligence were observed for any of the analyzed coping strategies. Across all models, the anxiety × emotional intelligence interaction terms were non-significant (all *p* > 0.10), indicating the absence of moderation effects.

Post-therapy assessment (T2)

For state anxiety at Time 2 (STAI1_T2), no significant moderating effects of emotional intelligence were found for any coping strategy. Most models were non-significant or showed non-significant interaction terms.

A different pattern emerged for trait anxiety at Time 2 (STAI2_T2). In the case of behavioral disengagement, a significant moderating effect of emotional intelligence was observed, such that higher emotional intelligence strengthened the positive association between trait anxiety and behavioral disengagement (Table 3). The observed moderation effect should not be interpreted as a stable maladaptive role of emotional intelligence.

A similar but weaker pattern (moderation trend) was observed for denial, where the association between trait anxiety and denial was significant only at average and high levels of emotional intelligence (Table 4).

No significant moderating effects were found for self-blame or self-distraction at Time 2.

Summary of moderation analyses (Table 5).

Overall, the findings suggest that emotional intelligence does not consistently serve a protective function in the context of anxiety-related coping. Under conditions of elevated trait anxiety, particularly after therapy, higher emotional intelligence may increase vulnerability to avoidance-oriented coping strategies, strengthening the association between experienced distress and withdrawal-related behaviors. This pattern may be interpreted as a cost of heightened emotional awareness when the capacity for effective affect regulation is limited.

### 3.4. Does Therapy Improve the Psychological Functioning of Patients Diagnosed with Personality Disorders?

The next stage of the analysis focused on verifying whether psychological therapy contributed to a reduction in anxiety levels. It was hypothesised that therapy would lead to a decrease in state anxiety in particular, given its sensitivity to situational and therapeutic influences.

Although the descriptive results suggested a decline in state anxiety scores from pre- to post-treatment, the statistical test did not confirm the significance of this change. A paired-samples *t*-test yielded the following result: t(29) = 1.853, *p* = 0.037, with a small-to-medium effect size (d = 0.338; 95% CI [–0.033, 0.704]).

Importantly, the confidence interval includes zero, indicating notable uncertainty around the true magnitude of the effect. Thus, while the direction of change is consistent with clinical expectations, the evidence remains statistically weak and should be interpreted with caution.

In contrast, trait anxiety remained stable, showing no meaningful differences between measurements: t(29) = −0.137, *p* = 0.892, d = −0.025. This result indicates that therapy did not affect participants’ more enduring anxiety tendencies, which is consistent with the theoretical understanding of trait anxiety as a relatively stable individual characteristic.

Together, these findings suggest that although a downward trend in state anxiety is visible, the available data do not allow us to conclude that therapy resulted in a statistically reliable reduction in anxiety symptoms. Future studies with larger samples may help clarify whether the observed trend reflects a true therapeutic effect or random variation (Table 6).

Next, it was examined whether a significant increase in emotional intelligence could be observed following psychological therapy, compared to the pre-treatment measurement. To this end, a paired-samples *t*-test was conducted again. The analyses revealed no statistically significant differences in the participants’ emotional functioning. Therapy did not lead to a change in the level of emotional intelligence.

Across coping strategies, the largest and most robust pre–post increases were observed for seeking emotional support, humour, and religion (all *p* < 0.001; medium-to-large effects). A trend-level reduction was found for self-blame (*p* = 0.072), whereas substance use did not change significantly (Table 7).

To examine changes in coping strategies over the course of therapy, paired-samples *t* tests were conducted comparing pre-treatment (Time 1) and post-treatment (Time 2) scores across adaptive and maladaptive coping strategies. Given potential deviations from normality, the robustness of results was additionally evaluated using bootstrap resampling (1000 samples), and effect sizes were estimated using Cohen’s *d* and Hedges’ *g* [21].

Overall, the results indicated no statistically significant pre–post differences for the majority of coping strategies, suggesting relative stability of coping patterns across the therapeutic period. No significant changes were observed for active coping, planning, seeking instrumental support, positive reframing, acceptance, denial, self-distraction, self-blame, behavioral disengagement, or venting (*p* > 0.10). Bootstrap-based confidence intervals for mean differences consistently included zero, further confirming the absence of robust pre–post change effects for these strategies.

For all remaining coping strategies, effect sizes were small in magnitude (|*d*| < 0.31), with 95% confidence intervals encompassing zero, indicating that any observed numerical changes were minor and likely attributable to sampling variability rather than systematic therapeutic effects.

Taken together, these findings suggest that psychological therapy was not associated with broad, uniform changes in coping strategy use, but may have contributed to a selective reduction in substance-use coping, a particularly maladaptive behavioral strategy. The overall stability of most coping strategies aligns with the conceptualization of coping styles as relatively trait-like and resistant to short-term change. At the same time, the observed decrease in substance use may reflect therapy-related improvements in behavioral self-regulation or reductions in reliance on avoidance-based coping mechanisms.

This pattern underscores the importance of distinguishing between behaviorally anchored coping strategies, which may be more amenable to therapeutic modification, and cognitive or dispositional coping tendencies, which may require longer-term or more targeted interventions to change meaningfully.

To examine changes in emotional intelligence and anxiety levels across the course of therapy, paired-samples *t* tests were conducted comparing pre-treatment (Time 1) and post-treatment (Time 2) scores. Effect sizes were estimated using Cohen’s *d* and Hedges’ *g*, with 95% confidence intervals.

The analyses indicated no statistically significant changes in emotional intelligence across the therapeutic period. Neither the overall emotional intelligence score (INTE total), *t*(29) = −0.08, *p* = 0.935, *d* = −0.02, 95% CI [−0.37, 0.34], nor its two components—Factor I (emotion recognition and understanding), *t*(29) = 1.15, *p* = 0.259, *d* = 0.21, 95% CI [−0.15, 0.57], and Factor II (emotion regulation), *t*(29) = 0.32, *p* = 0.753, *d* = 0.06, 95% CI [−0.30, 0.42]—showed significant pre–post differences. The associated effect sizes were small, and all confidence intervals included zero, indicating stability of emotional intelligence over time.

In contrast, a statistically significant reduction in state anxiety was observed following therapy. Scores on STAI X1 (state anxiety) decreased from pre- to post-treatment, *t*(29) = 1.85, *p* = 0.037, with a small-to-moderate effect size (*d* = 0.34, 95% CI [−0.03, 0.70]). This finding suggests that therapy was associated with a meaningful reduction in situational anxiety symptoms.

No significant change was found for trait anxiety (STAI X2), *t*(29) = −0.14, *p* = 0.892, *d* = −0.03, 95% CI [−0.38, 0.33], indicating that dispositional anxiety remained stable across the treatment period.

Taken together, these results suggest that therapy was effective in reducing state-dependent anxiety, but did not produce measurable changes in trait anxiety or emotional intelligence over the observed timeframe. This pattern is consistent with theoretical models positing that state anxiety is more sensitive to short-term therapeutic interventions, whereas emotional intelligence and trait anxiety represent more stable, trait-like characteristics that may require longer-term or more targeted interventions to change meaningfully.

### 3.5. Summary of Results

The aim of the conducted statistical analyses was to verify the relationships between the level of anxiety, emotional intelligence, and stress coping strategies, as well as to assess the effectiveness of psychological therapy in relation to the variables under study.

The hypothesis regarding the impact of therapy was partially confirmed—state anxiety was significantly reduced following therapy, whereas trait anxiety remained unchanged. These findings suggest that the therapeutic intervention may have had a beneficial effect on the temporary experience of anxiety, without altering the participants’ general emotional response patterns.

Correlations between anxiety levels and coping strategies revealed that higher anxiety was associated with more frequent use of maladaptive strategies, such as denial, and self-blame. Conversely, higher levels of emotional intelligence were positively correlated with adaptive strategies (e.g., active coping, planning), and negatively correlated with.

An important finding of the present study is that moderation effects of emotional intelligence emerged exclusively after therapy (T2) and only in relation to trait anxiety, whereas no such effects were observed prior to therapeutic intervention (T1). This pattern suggests that therapy may have altered the functional role of emotional intelligence in the anxiety–coping process, rather than simply reducing symptom levels.

Before therapy, emotional intelligence did not moderate the associations between anxiety and any of the avoidance-oriented coping strategies. This absence of moderation at T1 may indicate that, under conditions of elevated and relatively unprocessed anxiety, emotional resources are either insufficiently accessible or functionally overshadowed by distress intensity. In such a state, coping responses may be driven primarily by anxiety itself, leaving little room for individual differences in emotional competencies to shape coping patterns.

Following therapy, a different configuration emerged. In the post-therapy assessment, emotional intelligence moderated the relationship between trait anxiety and behavioral disengagement, and a similar, albeit weaker, pattern was observed for denial. Notably, higher emotional intelligence amplified rather than attenuated the association between trait anxiety and avoidance-oriented coping. This finding challenges the common assumption that emotional intelligence uniformly serves a protective or buffering role.

One plausible interpretation is that therapy increased emotional awareness and introspective capacity, particularly in individuals with higher emotional intelligence. As a result, participants may have become more sensitive to the presence and persistence of trait-like anxiety, which is typically more stable and less amenable to short-term change than state anxiety. In this context, heightened emotional insight may paradoxically intensify the subjective salience of anxiety-related distress, especially when full regulatory mastery has not yet been achieved.

From this perspective, emotional intelligence may function as a “double-edged resource” after therapy. While increased emotional awareness is a core therapeutic goal, it may initially expose individuals—especially those high in emotional intelligence—to a more vivid experience of unresolved anxiety. When adaptive regulation strategies are still developing, this heightened awareness may promote withdrawal-oriented responses, such as behavioral disengagement or denial, as short-term attempts to manage emotional overload.

Importantly, this interpretation aligns with models distinguishing emotional awareness from effective emotion regulation. Therapy may enhance the former earlier or more rapidly than the latter, creating a transitional phase in which individuals are highly aware of their emotional states but not yet fully equipped to regulate them adaptively. In such conditions, avoidance-oriented strategies may temporarily increase, particularly among individuals with higher emotional intelligence who are more attuned to internal states.

The fact that moderation effects were observed only for trait anxiety and not for state anxiety further supports this interpretation. Trait anxiety reflects a more enduring vulnerability, likely requiring longer or more targeted therapeutic work. After therapy, individuals high in emotional intelligence may recognize the chronic nature of their anxiety more clearly, which could contribute to disengagement or denial when perceived coping demands exceed current regulatory capacity.

Overall, the emergence of moderation effects exclusively at T2 suggests that therapy may reorganize the interplay between anxiety, emotional intelligence, and coping, rather than producing linear symptom reduction alone. These findings underscore the importance of considering post-therapy transitional processes, in which increased emotional insight may temporarily coexist with maladaptive coping patterns. Future research should examine whether these effects represent a transitional stage toward more adaptive regulation or a subgroup-specific vulnerability requiring tailored therapeutic support.

In summary, the results suggest the following:

There is a relationship between anxiety and coping strategies, particularly with respect to maladaptive strategies.
Emotional intelligence is associated with more beneficial, adaptive coping strategies.
Therapy had a positive effect on state anxiety.
Emotional intelligence does not have a stable, protective, moderating function. However, it may conditionally alter the anxiety-coping relationship after therapy.

## 4. Discussion and Conclusions

The findings of the present study provide valuable insights into the relationships between anxiety levels, emotional intelligence, and coping strategies. The analyses also allow for reflection on the effectiveness of psychological therapy and the moderating role of emotional intelligence.

One of the key findings is the statistically significant reduction in state anxiety following psychological therapy. This finding is consistent with the literature indicating that therapeutic interventions—particularly those based on the cognitive-behavioral approach—are effective in reducing symptoms of situational anxiety [22]. Such anxiety is dependent on current experiences and context, and is therefore susceptible to modification through psychoeducation, relaxation techniques, exposure, or cognitive restructuring.

The absence of significant differences in trait anxiety suggests that short-term therapy may be insufficient to bring about lasting changes in deeply rooted emotional tendencies. According to Spielberger’s conceptualization (1983), trait anxiety is a relatively stable component of personality; thus, its modification may require a longer duration of intervention, more individualized therapeutic work, or systemic approaches [5]. It is also worth emphasizing the importance of effect sizes, which—in a sample this small—offer more informative insight than *p*-values alone. In several areas, particularly those related to coping strategies, moderate or even large effects emerged, despite the wide confidence intervals. The strongest effects concern the increased use of humor, religion, and emotional support after the intervention, which may suggest a meaningful clinical shift, even if this requires confirmation in larger samples.

Moderate effects observed in the associations between anxiety and avoidant coping indicate that, even when significance tests are inconclusive, it is still worthwhile to consider psychologically meaningful tendencies that may be emerging in the data. This type of information helps identify which variables may represent the most promising targets for future research and interventions, and which ones require further verification using larger, more statistically stable samples.

Consistent with the hypotheses and previous research [23,24,25], individuals with higher levels of anxiety are more likely to employ maladaptive strategies such as self-distraction denial, and self-blame. While these strategies may reduce tension in the short term, over time they contribute to the maintenance of symptoms and a decline in psychosocial functioning.

A potential mechanism underlying this relationship may involve the limited availability of cognitive resources in individuals experiencing high levels of anxiety [26]. Anxiety consumes attentional capacity, impairs situational analysis, and hinders the planning of effective actions. As a result, such individuals are less likely to utilize task-oriented and more adaptive strategies.

Conversely, the correlation results revealed that higher levels of emotional intelligence are associated with more frequent use of task-oriented strategies, such as active coping and planning, and less frequent use of avoidant strategies. This finding is consistent with the research of Mortillaro et al (2023), as well as subsequent work by Coronado—Maldonado et al 2023 and O’Connor et al 2019 who defined emotional intelligence as the ability to recognize, regulate, and utilize emotions—both one’s own and those of others—in a way that promotes adaptation [9,27,28].

In the context of coping, emotional intelligence may function as a resource mechanism [29] that facilitates the selection of effective strategies. Emotionally intelligent individuals are able to recognize the source of stress more quickly, assess its impact, and select an appropriate course of action. As a result, they are less susceptible to impulsive, automatic anxiety-driven responses.

Although it was hypothesized that emotional intelligence might function as a moderator between anxiety and coping strategies, the analyses did not confirm this relationship. The lack of significant interaction effects may be attributable to several factors:

A limited sample size, which reduces the statistical power of interaction tests [30].

A possible lack of variance in the moderating variable within the sample (e.g., uniformly high levels of emotional intelligence).

Emotional intelligence may serve as a mediator rather than a moderator, i.e., it may mediate the relationship between anxiety and the choice of coping strategies—a possibility worth exploring in future research.

Finally, moderation may only emerge in specific subgroups (e.g., differing by age, educational level, or attachment style). Therefore, the absence of a moderation effect should not be interpreted as contradictory to theoretical assumptions, but rather as an indication of the need for further research—particularly studies with larger sample sizes and models that incorporate social context, life experiences, and other personality resources.

In light of the analyses conducted, the results provide empirical support for the role of emotional and cognitive resources in coping with stress. Consistent with earlier findings [9,29,31], emotional intelligence promotes the use of task-oriented strategies and reduces reliance on avoidant strategies, confirming its adaptive nature. The results also demonstrate that psychological therapy—particularly cognitive-behavioral in nature—can effectively reduce state anxiety [22], although it does not significantly impact trait anxiety, in line with its conceptualization as a relatively stable personality trait [5]. The observed correlations between anxiety and coping strategies corroborate earlier assertions [23,24] that individuals experiencing higher levels of anxiety tend to adopt maladaptive strategies, which may stem from limited cognitive resources under high stress conditions [26]. These findings indicate that individuals with higher emotional intelligence demonstrate greater capacity for effective emotion recognition and regulation, which translates into more effective stress management [27]. Although the existence of a moderating effect of emotional intelligence on the relationship between anxiety and coping strategies was hypothesized, the statistical analyses did not confirm this relationship.

Possible explanations include the limited sample size [30] low variability in emotional intelligence levels within the studied group, or the possibility that emotional intelligence functions more as a mediator than a moderator. These conclusions indicate the need for further research using more diverse samples and models that incorporate contextual and personality-related factors.

From a practical perspective, these results highlight the importance of supporting the development of emotional intelligence within preventive programs, psychological interventions, and therapeutic processes—particularly among individuals exhibiting high levels of anxiety. Strengthening these competencies may contribute to the adoption of more adaptive coping strategies and to improved overall psychological functioning.

The demographic structure of the sample (N = 30), while allowing for analysis of the group as a whole as a clinical sample, does not permit reliable subgroup comparisons, such as by gender or specific diagnoses. Internal heterogeneity, small sample size, and gender imbalance limit the generalizability of the findings and the feasibility of conducting differential analyses.

The results obtained should therefore be regarded as exploratory and pilot in nature, rather than fully conclusive. Further research in this area—particularly involving larger and more diverse samples, including variation in gender and personality disorder types—would allow for more representative results. Achieving gender balance and increasing sample size would also enable more advanced differential analyses and support more meaningful conclusions with clinical applicability.

## Figures and Tables

**Table 1:** Pearson correlations between emotional intelligence (INTE) and adaptive coping strategies at baseline (N = 30), with 95% BCa bootstrap confidence intervals. Time 1.

Coping Strategy	INTE Total *r* (95% BCa CI)	*p*	INTE Factor I—Emotion Recognition and Understanding *r* (95% BCa CI)	*p*	INTE Factor II—Emotion Regulation *r* (95% BCa CI)	*p*
Active coping	0.53 [0.18, 0.75]	0.002	0.68 [0.45, 0.81]	<0.001	0.30 [−0.15, 0.62]	0.108
Planning	0.62 [0.37, 0.81]	<0.001	0.69 [0.48, 0.85]	<0.001	0.47 [0.11, 0.70]	0.009
Seeking instrumental support	0.33 [−0.00, 0.60]	0.077	0.47 [0.11, 0.72]	0.010	0.04 [−0.31, 0.38]	0.853
Seeking emotional support	0.49 [0.15, 0.75]	0.006	0.50 [0.13, 0.79]	0.005	0.15 [−0.27, 0.50]	0.442
Positive reframing	0.53 [0.17, 0.77]	0.002	0.61 [0.27, 0.81]	<0.001	0.16 [−0.29, 0.55]	0.393
Acceptance	0.35 [0.00, 0.65]	0.055	0.34 [0.00, 0.62]	0.065	0.23 [−0.34, 0.65]	0.225
Humor	−0.05 [−0.39, 0.32]	0.789	−0.01 [−0.34, 0.35]	0.942	−0.23 [−0.56, 0.09]	0.214

**Table 2:** Pearson correlations between emotional intelligence (INTE) and adaptive coping strategies at post-treatment (Time 2). Bold text denotes statistically significant results (*p* < 0.05).

Coping Strategy	INTE Total *r* (95% CI)	*p*	INTE Factor I (Emotion Recognition and Understanding) *r* (95% CI)	*p*	INTE Factor II (Emotion Regulation) *r* (95% CI)	*p*
**Active coping**	0.35 [0.03, 0.64]	0.056	**0.44 [0.15, 0.66]**	**0.014**	0.09 [−0.22, 0.44]	0.629
**Planning**	**0.63 [0.31, 0.84]**	**<0.001**	**0.57 [0.29, 0.76]**	**<0.001**	**0.53 [0.10, 0.79]**	**0.003**
**Acceptance**	**0.40 [0.06, 0.63]**	**0.029**	0.34 [−0.01, 0.57]	0.066	**0.40 [0.00, 0.67]**	**0.028**
**Humour**	−0.14 [−0.44, 0.16]	0.471	−0.06 [−0.39, 0.33]	0.770	−0.16 [−0.49, 0.12]	0.400
**Positive reframing**	**0.53 [0.23, 0.77]**	**0.003**	**0.60 [0.33, 0.79]**	**<0.001**	0.29 [−0.10, 0.58]	0.117
**Seeking emotional support**	0.30 [−0.11, 0.70]	0.102	0.32 [−0.08, 0.64]	0.089	0.14 [−0.24, 0.50]	0.468
**Seeking instrumental support**	0.17 [−0.26, 0.58]	0.375	0.19 [−0.27, 0.55]	0.322	0.09 [−0.30, 0.46]	0.622

**Table 3:** Conditional effects of trait anxiety on behavioral disengagement at different levels of emotional intelligence.

Level of EI	Effect X → Y (β)	SE	t	*p*	95% CI
Low EI (−1 SD)	0.066	0.029	2.26	0.033	[0.006, 0.126]
Mean EI	0.111	0.019	5.99	<0.001	[0.073, 0.149]
High EI (+1 SD)	0.137	0.026	5.31	<0.001	[0.084, 0.190]

**Table 4:** Conditional effects of trait anxiety on denial at different levels of emotional intelligence.

Level of EI	Effect X → Y (β)	SE	t	*p*	95% CI
Low EI (−1 SD)	0.003	0.050	0.06	0.951	[−0.100, 0.107]
Mean EI	0.089	0.032	2.80	0.010	[0.024, 0.154]
High EI (+1 SD)	0.140	0.044	3.15	0.004	[0.049, 0.231]

**Table 5:** Results of moderated regression analyses (PROCESS Model 1) across two measurement points.

Time	Anxiety Variable (X)	Coping Strategy (Y)	Model F (df)	R^2^	X × EI (β)	*p*	Conclusion
T1	STAI1_1 (state)	Self-blame	1.77 (3,26)	0.17	−0.001	0.592	No moderation
T1	STAI1_1 (state)	Behavioral disengagement	4.22 (3,26)	0.33	−0.001	0.423	No moderation
T1	STAI1_1 (state)	Denial	2.00 (3,26)	0.19	0.002	0.106	Trend (ns)
T1	STAI1_1 (state)	Self-distraction	2.49 (3,26)	0.22	−0.0003	0.806	No moderation
T1	STAI2_1 (trait)	Self-blame	4.01 (3,26)	0.32	−0.0002	0.892	No moderation
T1	STAI2_1 (trait)	Behavioral disengagement	4.55 (3,26)	0.34	≈0	0.997	No moderation
T2	STAI1_2 (state)	Self-blame	2.20 (3,26)	0.20	0.0003	0.838	No moderation
T2	STAI1_2 (state)	Behavioral disengagement	7.69 (3,26)	0.47	0.001	0.577	No moderation
T2	STAI1_2 (state)	Denial	1.75 (3,26)	0.17	0.003	0.122	No moderation
T2	STAI1_2 (state)	Self-distraction	1.74 (3,26)	0.17	−0.001	0.578	No moderation
T2	STAI2_2 (trait)	Self-blame	5.96 (3,26)	0.41	0.002	0.429	No moderation
T2	STAI2_2 (trait)	Behavioral disengagement	12.61 (3,26)	0.59	0.002	0.097	Moderation (amplifying)
T2	STAI2_2 (trait)	Denial	3.49 (3,26)	0.29	0.004	0.065	Moderation trend
T2	STAI2_2 (trait)	Self-distraction	2.26 (3,26)	0.21	0.0004	0.813	No moderation

**Table 6:** A paired-samples *t*-test was conducted using repeated measures for anxiety as a state and as a trait before and after therapy.

		Mean	Standard Deviation
Pair 1	State anxiety before therapy	54.0333	13.39450
	State anxiety after therapy	47.4667	14.30152
Pair 2	Trait anxiety before therapy	55.9333	8.92472
	Trait anxiety after therapy	56.2667	10.87209

**Table 7:** A paired-samples *t*-test was conducted using repeated measures for coping strategies (1—before therapy, 2—after therapy). Bold text denotes statistically significant results (*p* < 0.05).

Pair	Variable	*t*(29)	*p*	Cohen’s *d*	95% CI *d*	Hedges’ *g*	95% CI *g*
1	INTE total	−0.08	0.935	−0.02	[−0.37, 0.34]	−0.02	[−0.36, 0.33]
2	INTE Factor I (emotion recognition and understanding)	1.15	0.259	0.21	[−0.15, 0.57]	0.21	[−0.15, 0.56]
3	INTE Factor II (emotion regulation)	0.32	0.753	0.06	[−0.30, 0.42]	0.06	[−0.29, 0.41]
4	**STAI X1 (state anxiety)**	**1.85**	**0.037**	**0.34**	**[−0.03, 0.70]**	**0.33**	**[−0.03, 0.69]**
5	STAI X2 (trait anxiety)	−0.14	0.892	−0.03	[−0.38, 0.33]	−0.02	[−0.37, 0.32]
6	Active coping	−0.80	0.428	−0.15	[−0.51, 0.21]	−0.14	[−0.49, 0.21]
7	Planning	0.65	0.522	0.12	[−0.24, 0.48]	0.12	[−0.24, 0.46]
8	Seeking instrumental support	−1.51	0.143	−0.28	[−0.64, 0.09]	−0.27	[−0.62, 0.09]
9	**Seeking emotional support**	**4.38**	**<0.001**	**0.80**	**[0.38, 1.21]**	**0.78**	**[0.37, 1.17]**
10	Venting	0.53	0.601	0.10	[−0.26, 0.45]	0.09	[−0.26, 0.44]
11	Self-distraction	0.28	0.783	0.05	[−0.31, 0.41]	0.05	[−0.30, 0.40]
12	Positive reframing	−0.85	0.400	−0.16	[−0.52, 0.21]	−0.15	[−0.50, 0.20]
13	Denial	−0.59	0.558	−0.11	[−0.47, 0.25]	−0.11	[−0.45, 0.25]
14	Self-blame	−1.87	0.072	−0.34	[−0.71, 0.03]	−0.33	[−0.69, 0.03]
15	Substance use	0.09	0.932	0.02	[−0.34, 0.37]	0.02	[−0.33, 0.36]
16	**Humour**	**4.39**	**<0.001**	**0.80**	**[0.38, 1.21]**	**0.78**	**[0.37, 1.18]**
17	**Religion**	**10.18**	**<0.001**	**1.86**	**[1.26, 2.45]**	**1.81**	**[1.23, 2.38]**
18	Acceptance	−1.53	0.136	−0.28	[−0.64, 0.09]	−0.27	[−0.63, 0.09]
19	Behavioral disengagement	−0.99	0.332	−0.18	[−0.54, 0.18]	−0.18	[−0.53, 0.18]

## Data Availability

The data presented in this study are available on request from the corresponding author due to privacy and ethical reasons.

## Supplementary Materials

The following supporting information can be downloaded at: https://www.mdpi.com/article/10.3390/jcm15041583/s1, CARE Checklist.
