# Supplementary material for: Anxiety and Emotional Intelligence as Predictors of Coping with Stress in Patients with Personality Disorders—A Single-Arm Pre–Post Observational Study
Source: J Clin Med. 2026 Feb 17;15(4):1583. doi: 10.3390/jcm15041583 (PMC12942224; doi:10.3390/jcm15041583)
Supplement: Supplementary file 1 [file jcm-15-01583-s001.zip › jcm-4018275-supplementary.pdf]

CARE case report guidelines

# CARE Checklist of information to include when writing a case report

(cc) BY-NC-ND

| Topic | Item | Checklist item description | Reported on Line |
|---|---|---|---|
| Title | 1 | The diagnosis or intervention of primary focus followed by the words "case report" | 3-4 |
| Key Words | 2 | 2 to 5 key words that identify diagnoses or interventions in this case report, including "case report" | 34 |
| Abstract (no references) | 3a | Introduction: What is unique about this case and what does it add to the scientific literature? | 15-19 |
| | 3b | Main symptoms and/or important clinical findings | 19-27 |
| | 3c | The main diagnoses, therapeutic interventions, and outcomes | 19-27 |
| | 3d | Conclusion—What is the main "take-away" lesson(s) from this case? | 28-33 |
| Introduction | 4 | One or two paragraphs summarizing why this case is unique (**may include references**) | 42-100 |
| Patient Information | 5a | De-identified patient specific information. | 113-117 |
| | 5b | Primary concerns and symptoms of the patient. | 113-117 |
| | 5c | Medical, family, and psycho-social history including relevant genetic information | 113-117 |
| | 5d | Relevant past interventions with outcomes | 113-117 |
| Clinical Findings | 6 | Describe significant physical examination (PE) and important clinical findings. | 152-172 |
| Timeline | 7 | Historical and current information from this episode of care organized as a timeline | 152-172 |
| Diagnostic Assessment | 8a | Diagnostic testing (such as PE, laboratory testing, imaging, surveys). | 152-172 |
| | 8b | Diagnostic challenges (such as access to testing, financial, or cultural) | 152-172 |
| | 8c | Diagnosis (including other diagnoses considered) | 152-172 |
| | 8d | Prognosis (such as staging in oncology) where applicable | 152-172 |
| Therapeutic Intervention | 9a | Types of therapeutic intervention (such as pharmacologic, surgical, preventive, self-care) | 120-133 |
| | 9b | Administration of therapeutic intervention (such as dosage, strength, duration) | 120-133 |
| | 9c | Changes in therapeutic intervention (with rationale) | 120-133 |
| Follow-up and Outcomes | 10a | Clinician and patient-assessed outcomes (if available) | 173-567 |
| | 10b | Important follow-up diagnostic and other test results | 173-567 |
| | 10c | Intervention adherence and tolerability (How was this assessed?) | 173-567 |
| | 10d | Adverse and unanticipated events | 173-567 |
| Discussion | 11a | A scientific discussion of the strengths AND limitations associated with this case report | 571-653 |
| | 11b | Discussion of the relevant medical literature **with references**. | 571-653 |
| | 11c | The scientific rationale for any conclusions (including assessment of possible causes) | 571-653 |
| | 11d | The primary "take-away" lessons of this case report (without references) in a one paragraph conclusion | 571-653 |
| Patient Perspective | 12 | The patient should share their perspective in one to two paragraphs on the treatment(s) they received | 134-150 |
| Informed Consent | 13 | Did the patient give informed consent? Please provide if requested 663 | Yes ☒ No ☐ |
